# Concours

**Published:** 1851-09

**Authors:** 


*Concours — Election of M. Nelaton.* — M. Nelaton, one of the surgeons to St. Louis, was elected Professor of Surgery to the Faculty of Medicine, on Wednesday last, and thus occupies the chair once filled with so much *eclat* by the illustrious Margolin. The election, it must be confessed, has occasioned universal surprise, for public opinion had selected another and more deserving candidate. The final decision was not arrived at without considerable hesitation,— one might almost say, without compromise of principle. It required no less than four different ballotings to arrive at a result. On the two first, the votes were thus distributed:

For M. Michon, four votes, viz., Bouillaud, Larrey, and Hervez de Chegoin.

For M. Buisson, three, viz., Velpeau, Begin, and Reveille Parise.

For M. Nelaton, three, viz., Rostan, Denonvilliere, and Langier.

For M. Robert, two, viz., Malgaigne, Gimella.*

On the third balloting, M. Robert's two voters went over to M. Nelaton; and, on the final *scrutin* between MM. Nelaton and Michon, the former was elected by a majority of seven to five votes.

The election, as I have said, has produced no small degree of surprise and regret, for the superiority of M. Buisson over all the other candidates was incontestable,— and it is seldom that the result of a Concours disappoints the judgment of the public. But, in the present case, it could hardly have been otherwise. M. Buisson was a stranger to the school of Paris, that is to say, pupil, representative, and professor of a rival institution; and his election would have been a virtual acknowledgment of the decadence of Parisian surgery. The schools of Paris and Montpelier have ever been opposed to each other, both in doctrine and educational practice, and *amour propre*,— a sentiment which influences even the most eminent men,— has, in the present

---

* The number of judges in a Concours is fifteen ; but three have been compelled to retire from ill-health, viz., MM. Roux, Cloquet, and Gerdy.

instance, given rise to an act of manifest injustice. This opinion is entertained by a vast majority of those who assisted at the Concours, though many allege, in justification, that the judges could not have acted otherwise.

M. Nelaton, the new Professor, is a young man of very considerable promise, and, although the Faculty may not have obtained the services of the best candidate, it will, at all events, be certain of possessing one fully competent to fulfil his duties. This is the main argument in favor of election by Concours. You are always sure of securing a man perfectly able to fill his office, though he may not be the *most* able,—a result which our British system often fails to produce.—*Paris Correspondent Med. Times, 24th May,* 1851.

